# Spondylodiscitis Caused by* Enterobacter agglomerans*

**DOI:** 10.1155/2016/8491571

**Published:** 2016-12-29

**Authors:** Jayaweera Arachchige Asela Sampath Jayaweera, Mahen Kothalawala, Balachandran Devakanthan, Sinnappoo Arunan, Dinithi Galgamuwa, Manori Rathnayake

**Affiliations:** ^1^Department of Microbiology, Faculty of Medicine and Allied Sciences, Rajarata University, Saliyapura, Sri Lanka; ^2^Teaching Hospital, Kandy, Sri Lanka; ^3^Department of Microbiology, Teaching Hospital, Kandy, Sri Lanka

## Abstract

All over the globe, the incidence of vertebral infection is rising. Nowadays, compared to tuberculous variety, pyogenic spondylodiscitis incidence is high. The increase in the susceptible population and improved diagnostics summatively contributed to this. In clinical grounds, differentiation of pyogenic and tuberculous spondylodiscitis is well defined.* Enterobacter agglomerans* is a hospital contaminant and associated with infections in immunocompromised individuals and intravenous lines. It causes a wide array of infections.* Enterobacter agglomerans* spondylodiscitis is unusual and there are, around the globe, only less than 31 suspected cases that have been previously reported.* Enterobacter agglomerans *histology mimics tuberculous rather than pyogenic spondylodiscitis. A 65-year-old farming lady, while being in hospital, developed sudden onset spastic paraparesis with hyperreflexia. Later blood culture revealed* Enterobacter agglomerans* with 41-hour incubation in 99.9% probability from Ramel identification system. Her initial ESR was 120 mm/first hour. Isolate was susceptible to ciprofloxacin and intravenous followed with oral therapy shows a drastic ESR fall and improved clinical response. Differentiation of tuberculous and pyogenic spondylodiscitis is very much important in management point of view. Therefore, blood culture has a role in diagnosis of spondylodiscitis. ESR can be used as important inflammatory marker in monitoring the response to treatment. Retrospectively, ESR would aid in reaching a definitive diagnosis.

## 1. Introduction

In elderly patient, pyogenic spondylodiscitis is the main manifestation of haematogenous variety of osteomyelitis [1]. Around the globe, incidence of vertebral infection is rising. An increase in the susceptible population and the improved diagnostics summatively contributed to this [2].* Staphylococcus aureus* is the major cause for pyogenic haematogenous spondylodiscitis [3], which is common following surgical interventions. On the other hand,* Escherichia coli* act as the most common etiologic agent following spontaneous Gram-negative bacteremia [4].* Propionibacterium* and* Bacteroides* species are the most commonly reported causes for anaerobic spondylodiscitis [5].

In almost 50% of cases of spondylodiscitis, a distant focus of infection has been identified. The genitourinary tract (17%), skin and soft tissue (11%), intravascular devices (5%), gastrointestinal tract (5%), respiratory tract (2%), and oral cavity (2%) are common foci, while spondylodiscitis was reported in 12%, following infective endocarditis [6]. Blood culture is a simple method for identifying bacterial agents of spondylodiscitis, as the infection is mostly monomicrobial and often has a haematogenous source [7].

Spondylodiscitis following* Enterobacter agglomerans* species is unusual with, around the globe, only 31 suspected cases having been previously reported. However, organism was not isolated in all cases [8];* Enterobacter agglomerans* causes bacteremia, lower respiratory tract infections, skin and soft-tissue infections, urinary tract infections (UTIs), endocarditis, intra-abdominal infections, septic arthritis, osteomyelitis, CNS infections, and ophthalmic infections in immunocompromised individuals and associated with intravenous lines [9].

We report a patient with spontaneous T10-T11 spondylodiscitis presented to Sri Lankan tertiary care hospital following* Enterobacter agglomerans*.

## 2. Case Presentation

A 65-year-old farming lady admitted to the hospital having three-month history of on and off dysuria and renal colic. She was continuously afebrile. While being in hospital, she has developed sudden onset bilateral lower limb weakness and severe backache. There were no history of trauma and fall and no recent weight loss. Her apatite was good. There was no past or contact history of tuberculosis. Physical examination revealed tenderness to palpation over the tenth and eleventh thoracic vertebrae. The neurologic examination reveals spastic paraparesis with hyperreflexia. Her cranial neuron examination was normal and she is well oriented (Glasgow Coma Scale 15/15).

**Figure 1:**
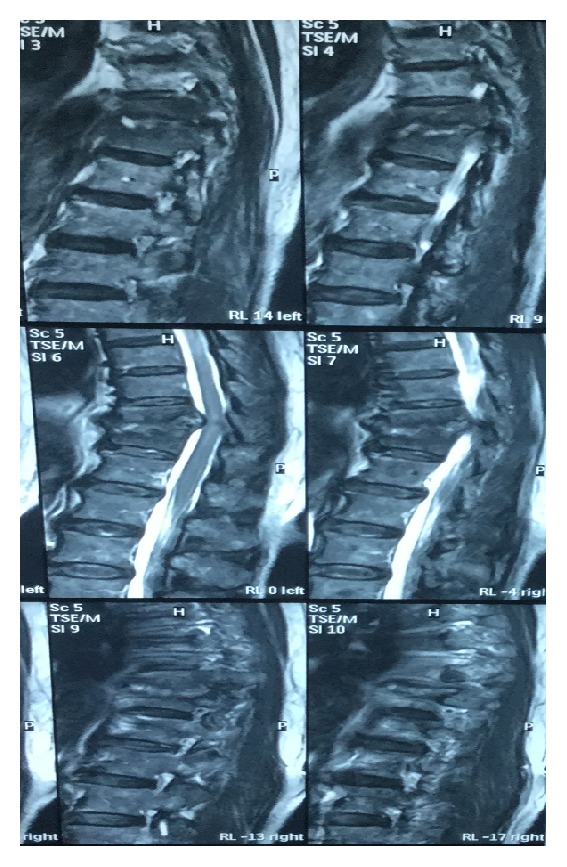
MRI thoracic spine T10-T11 discitis with minimal soft-tissue component and vertebral body.

**Figure 2:**
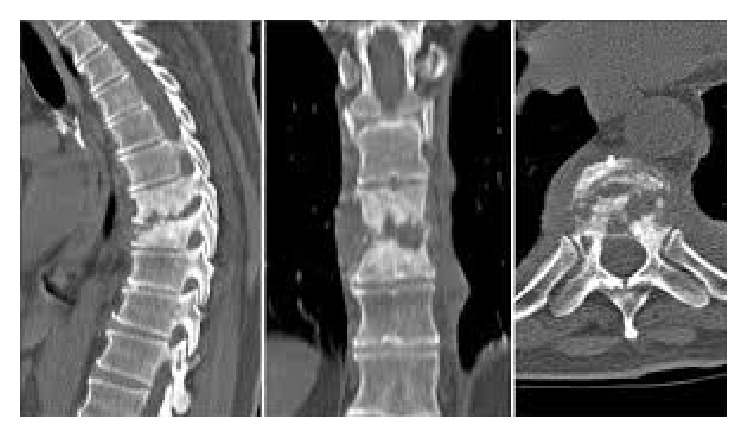
CT thoracic spine shows T10 vertebral body destruction.

Her total white cell count was 16,900/cmm (normal: 4.400–11,300/cmm) with 90.45% granulocytes, 5.6% lymphocytes, and 1.0% monocytes. Erythrocyte sedimentation rate (ESR) was 120 mm/hr. (normal: 0–10 mm/hr.). C-reactive protein (CRP) was 42 mg/dL (normal: <0.6 mg/dL). Magnetic resonance imaging (MRI) and CT of the thoracic spine revealed severe thoracic (T10-T11) discitis with minimal soft-tissue component and vertebral body destruction (Figures 1 and 2). Features are suggestive of Asian variety of tuberculous spondylodiscitis rather than pyogenic spondylodiscitis. No contiguous spread and no epidural and psoas muscle abscesses were found. So, we were in doubt to use the trial of antituberculous therapy or empiric antibiotic to cover common microbes causing pyogenic spondylodiscitis. Blood culture revealed* Enterobacter agglomerans* with 41-hour incubation in 99.9% probability from Ramel identification system. Isolate was susceptible to ciprofloxacin, carbapenems, and aminoglycosides and resistant to ceftazidime and aztreonam. We have started intravenous ciprofloxacin 400 mg 12 hourly with weekly monitoring of ESR and the clinical response (Table 1).

Computed topography- (CT-) guided biopsy culture was negative for bacteria, fungi, and tuberculosis. Histology shows possible bacterial infection. Chest X-ray radiography is normal and Mantoux test was negative.

**Figure 3:**
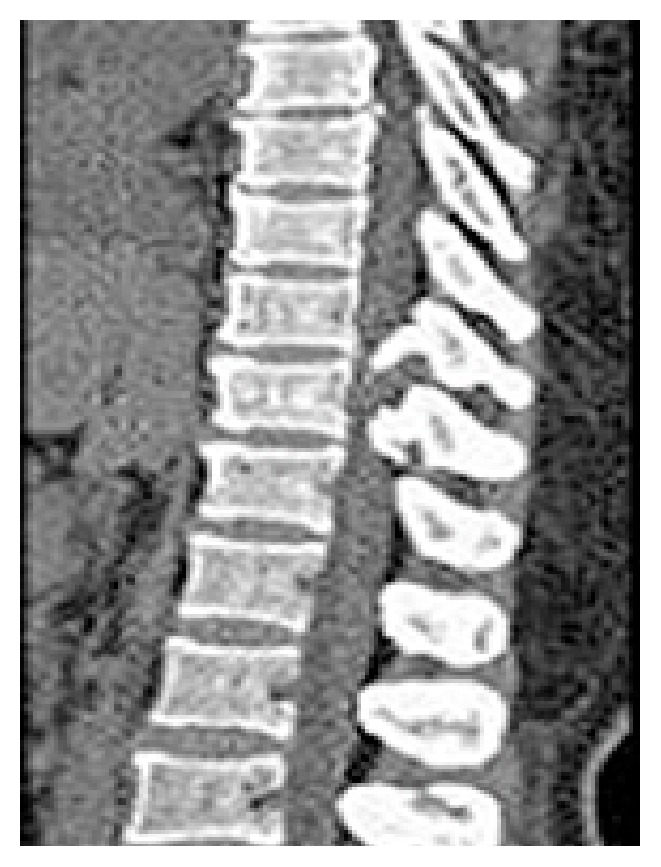
CT thoracic spine shows T10-T11 vertebral body and disc regeneration.

Imaging study of genitourinary system shows calculus over right renal pelvis. Urine culture was negative for bacteria. Patient was successfully treated with intravenous ciprofloxacin, 400 mg 12 hourly for 3 weeks followed with 3 weeks of oral ciprofloxacin. Follow-up examination on 6th week on completion of treatment showed mild residual bilateral lower limb weakness and ability to walk with aid. Follow-up CT in 6 weeks shows T10-T11 vertebral body and disc regeneration (Figure 3).

## 3. Discussion

In clinical grounds, differentiation of pyogenic and tuberculous spondylodiscitis is well defined. Further with the aid of microbiological and radiological evidence, diagnostic accuracy been well improved. In some instances, the clinical picture and microbiological and radiological evidence may mismatch, leading to dilemma in differentiation of pyogenic and tuberculous spondylodiscitis [10]. Such differentiation is utterly important in patient management; thus, in tuberculous spondylodiscitis, it requires minimal of 1-year antituberculous treatment [11]. On the other hand, in pyogenic spondylodiscitis, it requires a minimum of 6 weeks of targeted antimicrobial therapy. In both instances with targeted therapy in such time period, neurological sequela is reversible and they ended up with better outcome. On the contrary, often the diagnosis of spondylodiscitis is delayed and leads to poorer prognosis.

*Enterobacter agglomerans* is in family Enterobacteriaceae. It causes opportunistic human infections, mostly as wound infection following contamination of plant material and as hospital-acquired infections [12]. While in ward, this lady presented with sudden onset paraparesis. The sudden onset nature is leaning towards being pyogenic in origin. Tuberculous spondylodiscitis is commonly subacute in onset. Usually 46% of cases of pyogenic spondylodiscitis and only 17% of tuberculous spondylodiscitis have fever as a complaint [13]. Throughout this period, this lady was afebrile.

Also computed topography and magnetic resonance imaging studies show thoracic vertebral involvement with extensive destruction of T10 and T11 vertebral bodies and T10-T11 disc destruction. Tuberculous spondylodiscitis mainly affects thoracic and lumber vertebral columns and leads to extensive destruction of vertebral bodies, while pyogenic spondylodiscitis involves lumber vertebra and disc involvement with minimal vertebral body destruction seen. Usually, in pyogenic spondylodiscitis, anterior part of vertebra is involved, while, in tuberculous spondylodiscitis, posterior vertebra is involved. Further in tuberculous spondylodiscitis, paraspinal and psoas abscess associate [10]. Here, T10 and T11 vertebrae have extensive destruction that favors tuberculous spondylodiscitis. ESR value of 120 mm in the first hour favors tuberculous in origin. Spondylodiscitis following Gram-negative bacteria and* Mycobacterium tuberculosis* have some overlapping features. Gram-negative bacterial spondylodiscitis is more likely to be lower levels of inflammatory markers and less abscess formation [14]. Destruction of disc is common [15]. In this case, CT-guided biopsy for bacterial culture was negative. But histology favors bacterial infection. Convincingly,* Enterobacter agglomerans *histological features are suggestive of granulomatous inflammation rather than bacterial variety. So, cumulatively, this mixed picture is more towards favors in tuberculous spondylodiscitis. But tuberculous culture was found to be negative on follow-up. Since the patient was stable, there were no features of sepsis; we did the watchful waiting rather than commencing empiric antimicrobials or trial of antituberculosis treatment. Following* Enterobacter agglomerans *detection in blood culture, the diagnosis shifted towards pyogenic spondylodiscitis. Still blood culture became positive 41 hours following incubation and thus made us hesitant to declare it is as the potent etiological agent for pyogenic spondylodiscitis. On the other hand,* Enterobacter agglomerans* is rare as a potent pathogen in immunocompetent individuals. Further, it is usually a hospital contaminant.

While in ward, she developed the weakness that would lean more towards nosocomial rather than occupational acquisition. This lady had 3-month history of on and off dysuria associated with renal calculus.* Enterobacter agglomerans* pyelonephritis and urinary tract infections have been described, but association with renal calculus is been not described. This lady did not have clinical features of pyelonephritis, but urine full reports show 100–150 pus cells in high power field and urine culture having no growth. Still the association of renal calculi with* Enterobacter agglomerans* could not be excluded.

Since initial diagnosis as pyogenic spondylodiscitis was doubtful, following with blood culture positivity of* Enterobacter agglomerans*, we have started trial of anti-antimicrobials rather than antituberculosis treatment. We have used ESR as prognostic marker of spondylodiscitis and, with the continuation of ciprofloxacin ESR, dramatically responded. Simultaneously, clinical response was seen as her paraparesis showing gradual improvement where lower limbs tend to move and with the completion of 6 weeks of treatment power it became 5/5 as she was capable of walking with aid. Subsequently, she became totally independent and returned to her day-to-day activities in total of 2 months. Throughout this period, she was on physiotherapy.

Blood culture has a role in the diagnosis of spondylodiscitis. Further, ESR can be used as an important inflammatory marker in monitoring the response to treatment. Retrospectively, ESR would aid in reaching a definitive diagnosis.

## Figures and Tables

**Table 1:** Clinical response and change of ESR with time following parenteral and oral ciprofloxacin treatment.

	Week 0	Week 1	Week 2	Week 3	Week 4	Week 5	Week 6
ESR mm/1st hr	120	100	80	70	26	12	13
Lower limb power	0/5	0/5	1/5	1/5	3/5	4/5	5/5
